# PROTOCOL: Mutual Help Organizations to Support Recovery Among Individuals Who Use Drugs: A Systematic Review Protocol

**DOI:** 10.1002/cl2.70021

**Published:** 2025-03-04

**Authors:** Emily A. Hennessy, Brandon G. Bergman, Leonard L. Levin, Jenny O'Connor, Morgan Klein, John F. Kelly

**Affiliations:** ^1^ Harvard Medical School, Recovery Research Institute Boston Massachusetts USA; ^2^ Francis A. Countway Library, Harvard Medical School Boston Massachusetts USA; ^3^ Recovery Research Institute Boston Massachusetts USA

**Keywords:** addiction recovery, drug use disorder, mutual‐help organizations, systematic review

## Abstract

This is the protocol for a Campbell systematic review. The objectives are as follows: (1) What kinds of mutual‐help organizations for drug use disorders have been studied with evidence available in the empirical literature? (2) What is the nature of the evidence with regard to the different types of mutual‐help organizations?—(a) To what extent are these entities shown to help initiate, sustain, and enhance rates of remission from drug use disorders and improve other functional outcomes when the available evidence is subjected to rigorous scientific scrutiny? (b) To what extent do different mutual‐help organizations confer differential benefit and to whom, how, and over what period? (c) To what extent have cost benefit or cost effectiveness studies indicated that these entities are cost‐effective?

## 1 Background

### 1.1 The Problem, Condition, or Issue

Drug use disorders (DUDs; e.g., opioid, cocaine, methamphetamine, cannabis) are prevalent psychiatric disorders particularly in high‐income countries (e.g., the United States, the United Kingdom, France, Australia; Degenhardt et al. 2013). They exact an enormous human and economic toll on individuals, families, and societies, conferring high disease burden and premature mortality. Most societies provide myriad forms of professionally delivered treatment including both inpatient and outpatient options to address drug use and addiction recovery, which have proven helpful (Prendergast et al. 2002). There are myriad ways of operationalizing addiction recovery that depend on the contexts of the individual defining the experience (e.g., person with lived experience, clinician, policy maker, scientist, etc.). For the purposes of this review, recovery is the process of resolving severe alcohol and other drug problems, addressing the consequences of these problems, and maintaining efforts to prevent the return of these problems.

Yet, despite the presence of programs for drug use treatment, individuals seek additional support after treatment, or do not want, or are not able to gain, access to formal treatment, and instead seek nonprofessional community‐based supports. Indeed, 12‐step groups are the second most common source of help after private physicians for DUD (Grant et al. 2016). Many societies around the world possess a variety of informal but highly ubiquitous resources run almost entirely by peers with lived experience of addiction and recovery from it. Typically known as “self‐help groups” or “mutual‐help organizations” (MHOs) these supports are designed to help individuals achieve long‐term remission and stable recovery from substance use disorder. Examples include those from the abstinence‐based, 12‐Step tradition (e.g., Narcotics Anonymous [NA], Cocaine Anonymous [CA], or Marijuana Anonymous [MA]) or from a growing array of non‐12‐step alternative MHOs (e.g., Self‐Management and Recovery Training [SMART] Recovery, Refuge Recovery/Recovery Dharma, LifeRing).

MHO's are typically free to the end user, but rely on voluntary donations to operate (Humphreys 2004; Kelly and Yeterian 2011). Their widespread availability in easily accessible locations both in‐person and online, mean that these entities have immense reach. They can also be accessed over the long‐term as needed, making them well‐suited to support individuals with DUDs which often produce continued biopsychosocial instability during the early years of remission. Similar to research on these organizations' demonstrated ability to help those alcohol use disorder (AUD) (e.g., Kelly et al. 2020), research suggests many individuals with other DUDs in addition to alcohol can attain stability and long‐term recovery through engaging with these recovery entities. Yet, despite this evidence, there is limited systematic empirical review of these organizations' ability to confer benefit to individuals suffering from DUDs to stop drug use or to help prevent relapse following index episodes of clinical care. With greater knowledge regarding the state of the science in this area, clinical practice paradigms could be updated and informed vis a vis the clinical utility of linkage to these groups, and policy could be constructed and implemented to create clinical paradigm shifts needed to incorporate this evidence.

### 1.2 The Intervention

MHOs are run by and for individuals seeking recovery from substance use disorder. They offer regular meetings for their members, where a key component is sharing personal experiences in active addiction and recovery with each other, providing opportunities for connection, and providing some form of social accountability to support the recovery journey. Different types of DUD recovery MHOs will offer different types of meeting options, structures, underlying philosophy and group norms, and ways to engage with other members. If in person, MHO meetings are often run in community spaces including, for example, churches, college campuses, and community centers, but virtual options are also becoming increasingly available.

MHOs based on 12‐step program philosophy of AA operate on the principles of anonymous membership and focus on abstinence from all substances, but do not require it. Meetings typically run for between 60 and 90 min (Kelly and McCrady 2008). Because of their ubiquitous nature, meetings can be found 7 days a week at almost any hour in many locations in person and now around the clock across the world. Although anyone is welcome to attend these meetings, they take an abstinence‐based approach and are geared towards and welcome individuals who express a willingness to stop drinking or using substances and are seeking support for stopping use (Zemore et al. 2017). At meetings, members are often encouraged to share their past and present experiences, although some meetings are structured to have only a single speaker, or a smaller limited number of speakers share their story (e.g., “speaker meetings”). A major focus of meetings is “the meeting before the meeting” and “the meeting after the meeting.” This is where informal connection including swapping contact information (e.g., phone numbers) can take place so that continued supportive connections can be made between the regular meetings (e.g., at times of high risk or emotional distress or to share a success). This social networking has proven to be a major therapeutic mechanism of behavior change within 12‐step MHOs (Kelly 2017; Kelly et al. 2012). Meetings often start with, and/or can be entirely focused on, a passage or chapter from 12‐step literature, such as the main text in NA or the 12‐step book (e.g., 12 Steps and 12 Traditions; Alcoholics Anonymous World Services Inc. 1952). A key component is obtaining a more experienced 12‐step recovery guide (e.g., sponsor or peer). Specifically, members are encouraged to work through the 12‐Steps of recovery with the aid of a 12‐step “sponsor” who is a more experienced member often with longer‐term recovery and utilizing such a person has been found to confer perhaps the biggest recovery benefits (Emrick et al. 1993). AA and NA both define sponsors as, primarily someone who has been through the 12 steps themselves, and has a good understanding of the recovery program as laid out in the steps. They are also willing to build special, supportive 1–1 relationships with the sponsee. They are typically more experienced members (Alcoholics Anonymous 2017; Narcotics Anonymous World Services 2004). There are also explicit spiritual elements interwoven throughout 12‐Step philosophy, which some members utilize while others do not (Kelly et al. 2012).

Furthermore, given the potential beneficial effects of engaging in 12‐step meetings, some treatments exist to facilitate an initial linkage with MHOs, often referred to as, 12‐Step Facilitation (TSF) interventions. The commonality across all the different modes of TSF interventions is education and discussion of what 12‐step MHOs are and what they do, the nature of 12‐step meetings and what to expect when attending, and different degrees of systematic encouragement or active linkage to attend 12‐Step meetings (Kelly et al. 2017). For example, depending on the study design, the TSF condition could range from a clinician simply providing brief advice all the way through proactive linkage to a current 12‐Step member who reaches out to the participant or even joins them at a meeting to multiple TSF sessions delivered weekly over several months.

Newer, non‐12‐step, or “second wave” MHO's operate on different principles and have different practices and emphases (Bergman et al. 2024). Because of their less‐established nature, they may not be as widely available and accessible as MHOs based on 12‐step program philosophy. Some of newer MHO's include reduced use/harm reduction as well as cessation goals and others omit any spiritual focus, which may be more appealing to individuals not seeking complete cessation, or who may be using medication to assist their recovery pathway (Monico et al. 2015). For example, the MHO SMART Recovery was developed from common clinically delivered motivational and cognitive and behavioral treatments and its members report choosing it for these specific scientifically focused foundations (Kelly et al. 2024). Unlike 12‐Step MHOs which have specific fellowships for individual drug types (e.g., CA for cocaine use disorder; NA for opioid use disorder; MA for cannabis use disorder, etc.) SMART Recovery addresses any type of substance use problem and abstinence or reduction goal. Additionally, the SMART meetings are operated by trained facilitators who do not have to have lived experience of addiction. During the meetings, the facilitators teach skills in a traditionally didactic format but also seek to facilitate interactions between members of the group.

Thus, there are now numerous other MHOs beyond the older and more ubiquitous 12‐step MHOs. All MHOs share common elements regarding their group format and meeting duration, focus on social support and camaraderie; sharing of experiences, challenges, and how to cope with these and stay in recovery. They vary in explicit content, focus, and emphases, regarding the etiological and maintaining factors pertaining to addiction disorders and the potential significance and role of other presumed therapeutic ingredients (e.g., SMART does not emphasize fellowship support outside of meetings, does not explicitly recommend a “Sponsor” equivalent who can assist new members, does not recommend long‐term or life‐long participation, and does not contain any spiritual focus, unlike 12‐step organizations).

### 1.3 How the Intervention Might Work

Each MHO possesses theoretical foundations regarding how therapeutic change is supposed to occur. Some MHOs are more explicit about these than others. In general, most MHOs view social network support and social network change as key to successful initiation and maintenance of remission and in the achievement of stable long‐term recovery from DUD. In turn, the social network and social ethos within meetings facilitates exchanges of recovery coping skills, helps instill hope and optimism that recovery is possible, and helps new members build new confidence and recovery self‐efficacy (Kelly et al. 2012). Existing research supports the notion that MHOs may confer recovery benefits through multiple mechanisms simultaneously (Kelly et al. 2012) and include the successful mobilization of many of these mechanisms listed above as well as in helping to reduce craving and impulsivity, and increase and maintain recovery motivation over time (Kelly 2017; Kelly et al. 2012). A main advantage of these community‐based MHOs is that they can provide long‐term, flexible, and widely available free access to robust communities of recovery support that can mobilize these mechanisms over time and facilitate enduring change. The long‐term risk of relapse and DUD reinstatement during the early months and years of recovery makes these entities a good match for the undulating course of early recovery.

It has also been found that, like formal treatments for DUDs, MHOs confer benefit differently for different people. Compared to men using AA, for example, women benefit just as much, but in different ways; women's ongoing recovery is helped by AA's ability to help them cope with negative affect without alcohol consumption whereas among men, AA helps prevent relapse by boosting men's confidence in coping with high risk social situations without resorting to alcohol use in situations where alcohol or other drugs may be present (Kelly and Hoeppner 2013). Also, among more severely addicted individuals, AA helps them more through boosting spirituality and thereby prevent relapse, whereas spirituality is not as important as the social network change factors among AA participants with less severe addiction histories (Kelly et al. 2012). In sum, MHOs appear to possess a variety of therapeutic offerings that participants maybe choose from to help them cope with the challenges related to their socio‐demographic characteristics, their life‐context and life‐stage, as well as their stage of recovery. These chosen therapeutic elements are also likely to change dynamically across stages of increasing recovery stability, emotional maturity, and neurophysiological repair (Eddie et al. 2022).

### 1.4 Why It Is Important to Do This Review

Most previous systematic reviews that have synthesized evidence on MHOs focused on those whose primary concern was related to alcohol use and AUDs. Alternatively, reviews that have focused on broader types of MHOs have done so to examine them against a single type of intervention program (e.g., compared to DBT; Giannelli et al. 2019) or for a specific population (e.g., for Indigenous peoples; Dale et al. 2019). Yet, one previous Campbell Review (Bøg et al. 2017) focused on one type of MHO, 12‐Step, for illicit DUDs. The review only included RCTs or quasi‐experimental studies with a well‐defined control group identifying 10 RCTs, 9 of which could be included in a meta‐analysis. Since that Campbell review, over 7 years ago, the newer types of MHOs have emerged and grown that address DUDs, and new studies have been published that would likely be available for synthesis. In addition, our review focuses on MHOs beyond just 12‐Step‐based organizations, including SMART recovery and Recovery Dharma and others, allowing for a broader perspective on the potential utility of MHOs. It will also allow for a nuanced examination of their relative fit and effectiveness for different individuals. The findings from this review could be used to support practice guidelines, as was evident from a similar review conducted by Kelly et al. (2020) on 12‐Step programs for AUDs. It could also be used to support policy decisions, such as those related to healthcare insurance coverage for extended recovery supports beyond treatment settings.

## 2 Objectives

1. What kinds of DUD recovery‐focused MHOs have been studied with evidence available in the empirical literature?
2. What is the nature of the evidence with regard to quantity and quality on the different types of MHOs?
  a. To what extent are these entities shown to help initiate, sustain, and enhance rates of remission from DUDs and improve other functional outcomes when the available evidence is subjected to rigorous scientific scrutiny?
  b. To what extent do different MHOs confer differential benefit and to whom, how, and over what period?
  c. To what extent have cost benefit or cost effectiveness studies indicated that these entities are cost‐effective?

## 3 Methods

The review methods will follow the Campbell Author Guidelines and utilize standardized reporting templates in line with Campbell review standards. The Title Registration Form for this review was submitted on June 10, 2024, to the Campbell Collaboration (“Mutual Help Organizations to Support Recovery among Individuals with Drug Use Disorders: A systematic review”) and approved on June 13, 2024.

### 3.1 Criteria for Considering Studies for This Review

#### 3.1.1 Types of Studies

Given the difficulties of conducting RCTs in recovery‐based services, which are outside the confines of formal treatment systems, we anticipate few RCTs of MHO's available for this review. Thus, both RCTs and non‐RCTs will be eligible for inclusion in the review to draw comparisons between study designs of varying rigor. Eligible types of non‐RCTs include quasi‐experimental prospective studies comparing two or more types of programming where at least one of the treatment programs/groups involves incorporating eligible mutual‐help group philosophy (e.g., TSF) or otherwise proactively prescribes involvement or facilitates linkage to addiction recovery MHOs during treatment. Studies that conduct cost‐effectiveness or cost–benefit analysis will also be included.

#### 3.1.2 Types of Participants

Individuals seeking to stop or reduce the use of drugs (other than alcohol and nicotine), including, but not limited to opioids, stimulants, and cannabis. Individuals in the study may also have an AUD but the majority of the sample (> 50%) should identify a drug other than alcohol as their primary substance from which they are seeking recovery (e.g., heroin, cocaine, cannabis, etc.). Given the developmental and programmatic differences between adolescents and adults seeking recovery (Hennessy et al. 2019), the sample of included studies must focus on individuals ages 18 or older. There is no maximum age limit of included samples for the review. If there is not enough information about the sample within the published paper to determine eligibility, we will reach out to authors for clarification and wait for their response for 30 days before making a decision about inclusion. If they follow‐up at a later point with the clarifying information, we will revise the screening decision, as necessary.

#### 3.1.3 Types of Interventions

Studies that focus on any of the following MHOs will be eligible for the review: Celebrate Recovery, CA, Crystal Methamphetamine Anonymous, Methadone Anonymous, LifeRing Secular Recovery, MA, NA, Refuge Recovery/Recovery Dharma, Secular Organizations for Sobriety, SMART Recovery, and Women for Sobriety.

Studies that examine the impact of TSF/MHO facilitation combined with treatment will also be eligible (e.g., Kelly et al. 2017), provided the MHO Facilitation approach involves facilitating access to a community‐based MHO (it cannot be located as part of/within the treatment center where the only access to it are through admitted clients).

If there is participation in multiple types of MHOs that are in addition to the organizations listed (e.g., Alcoholics Anonymous) within the sample, a majority of the sample (55%) must be attending one of the eligible organizations or have received a treatment that is proactively linking patients to one or more of these organizations.

#### 3.1.4 Types of Outcome Measures

##### 3.1.4.1 Primary Outcomes

In this review, we aimed to capture the widest range of recovery definitions, while remaining tethered to recovery as conceptualized in MHOs, the intervention of focus (see also White 2007). The scientific measures that map onto recovery are manifold, but we prioritize for inclusion those that are most commonly included in MHO research. Eligible studies will be those that examine and report at least one of the following outcomes: drug use frequency/quantity (e.g., percentage of days abstinent; percentage days intoxicated/impaired), proportion of individuals who are continuously abstinent, average longest period of abstinence, and addiction severity according to a validated measure (e.g., Leeds Dependence Questionnaire scores; Raistrick et al. 1994).

##### 3.1.4.2 Secondary Outcomes

Studies that report the following secondary outcomes will only be included if they report at least one of the predefined primary outcomes: cost‐effectiveness/health care cost‐offsets, employment, criminal justice involvement, indices of psychological functioning and well‐being (e.g., psychiatric symptoms such as depression and anxiety, happiness, self‐esteem), housing status, health status, quality of life and functioning, drug use consequences, and recovery capital scores (e.g., Assessment of Recovery Capital, Multidimensional Inventory of Recovery Capital; Bowen et al. 2023; Groshkova et al. 2013). These secondary outcomes were selected to focus on the key markers of recovery progress, as identified in a variety of recovery definitions and any reported will be collected.

##### 3.1.4.3 Duration of Follow‐Up

No specific duration of follow‐up will be required for eligibility.

##### 3.1.4.4 Types of Settings

No specific type of setting will be required for eligibility.

### 3.2 Search Methods for Identification of Studies

We will search a variety of electronic databases/hosts and websites for both published and unpublished literature with no restrictions on publication year. There will be no language restrictions in the search strategy to ensure inclusivity of relevant studies from all regions. However, we anticipate that a significant portion of studies may originate from English‐speaking countries, which reflect distinct cultural, policy, and healthcare contexts around substance use recovery. We will also consider and discuss findings from non‐English literature to ensure broader representation of global perspectives.

#### 3.2.1 Electronic Searches

See Supporting Information S2: Appendix for the full search strategy with terms for each electronic database. We will search the following databases: OVID Medline, Embase, CINAHL, Psycinfo, and Web of Science.

#### 3.2.2 Searching Other Resources

Additional searches will be conducted in NIH RePORTER and CENTRAL (*Cochrane database of trials*) to identify gray literature such as unpublished trials and conference abstracts. We will search the reference list of included studies and relevant reviews retrieved by the search. Experts will be contacted in an attempt to identify unpublished and ongoing studies. We will also hand‐search any key journals that return a high number of hits.

### 3.3 Data Collection and Analysis

#### 3.3.1 Description of Methods Used in Primary Research

Previous reviews on MHOs, such as the recent review of 12‐Step for AUD conducted by Kelly et al. (2020) and the previous Campbell Review (Bøg et al. 2017) of 12‐Step for illicit DUDs, included 21 and 10 RCTs/quasi‐RCTs respectfully. Given the narrow scope of these two reviews, and that our review will consider a wider breadth of MHOs for inclusion, we expect to find many eligible RCTs/quasi‐RCTs. Yet, due to the difficulty in randomizing individuals to community‐based support services, we also expect that some studies will employ nonrandomized, quasi‐experimental designs. The inclusion criteria outlined above requires nonrandomized trials to be prospective studies comparing two or more types of programming or otherwise proactively prescribing involvement with the intervention of focus.

In contrast, a cross‐sectional study seeking to describe the characteristics of MHO attendees, such as the recent study by Galanter et al. (2022) would not be considered eligible for this review based on the study design. In this study, the authors reported on outcomes among 2152 adults who were long‐term NA members. Participants completed a one‐time online survey that included questions about the effectiveness of virtual and in‐person NA meetings, the cohesion of NA members, and belief in the 12‐step programming. The survey does include questions to assess abstinence, but this is accomplished retrospectively and does not classify as a quasi‐experimental prospective design. Thus, it would not be eligible for the review, although it does make a significant contribution to our understanding of virtual 12‐step attendance in the context of the COVID‐19 pandemic.

#### 3.3.2 Selection of Studies

The deduplication and screening process will be conducted using Covidence Systematic Software (n.d.). Once duplicates are removed, all studies will be subject to the full screening process regardless of the search method used. First, two reviewers trained in review methods will read the title and abstracts to determine if studies are possibly eligible or clearly ineligible. Discrepancies will be resolved by discussion to reach consensus. The full text of studies included based on title/abstract screening will be retrieved for further screening. Once full texts are retrieved, two reviewers will independently screen the studies and make a final determination to include or exclude the study based on the eligibility criteria. Discrepancies will again be resolved by discussion to reach consensus; if there are any issues reaching consensus, a third reviewer will make the final determination. If eligibility cannot be determined based on the report, authors will be contacted to gather missing information. Any studies excluded at the full‐text level will be documented along with the reason for exclusion and any studies that meet the eligibility criteria will be included in the review. Outcomes of the search and screening process will be documented in a PRISMA flow chart (Moher et al. 2015).

#### 3.3.3 Data Extraction and Management

Two reviewers trained in review methods will be trained on the data extraction tool for this review using a training set of 2–5 studies. Following this training period, they will independently extract data from all included studies and will resolve any disagreements by discussion. During data extraction, all reviewers will follow the same coding guidelines that cover the following areas: population and demographics, intervention setting and description, comparator description, study design and follow‐up lengths, outcome constructs and results, funding sources and conflicts of interest (see Supporting Information S1: Appendix for a draft codebook). Discrepancies will be resolved by discussion to reach consensus.

#### 3.3.4 Assessment of Risk of Bias in Included Studies

Two reviewers will independently assess the quality of all included studies. If any included studies are authored by one of the reviewers, a different reviewer will assess quality to prevent a conflict of interest. Disagreements will be resolved by discussion to reach consensus.

RCTs will be assessed using the Cochrane Risk of Bias tool (RoB 2; Sterne et al. 2019). The Risk of Bias tool can be used to assess the methodological quality of any type of randomized trial based on five domains: the randomization process, deviations from intended interventions, missing outcome data, outcome measurement, and selection of the reported result. Given resource constraints, the risk of bias will only be examined for each primary (not secondary) outcomes. For the second domain (deviations from intended interventions), the RoB 2 tool requires authors to indicate the effect of principal interest at the protocol stage as either (1) the effect of assignment or (2) the effect of adherence. For this review, quality will be assessed using the effect of assignment to conduct an intention‐to‐treat analysis. Reviewers will complete a multistep process to assign a risk of bias rating for each domain. First, reviewers will answer a series of “signaling questions.” Based on the responses, reviewers will use the Cochrane algorithm to extract a risk of bias rating. Reviewers will have the opportunity to diverge from the predicted risk of bias judgment if they feel a different rating is more appropriate. For each domain, reviewers will ultimately assign one of three possible ratings: low risk of bias, some concerns, or high risk of bias.

Non‐RCTs will be assessed using the Effective Public Health Practice Project (EPHPP) Quality Assessment Tool for Quantitative Studies (Armijo‐Olivo et al. 2012). The EPHPP tool can be used to assess the methodological quality of both randomized and non‐randomized trials, but for the purpose of this review will be used only for nonrandomized trials. The quality assessment includes ratings for eight domains: selection bias, study design, confounders, blinding, data collection methods, withdrawals and dropouts, intervention integrity, and analysis. Reviewers complete a multistep process to assign one of three quality ratings for each domain: strong, moderate, or weak. For each domain, reviewers will answer a series of questions. Then reviewers will use the EPHPP Quality Assessment Dictionary to assign a rating based on their responses. Reviewers will use the individual domain ratings to assign a global methodological quality rating of strong, moderate, or weak.

#### 3.3.5 Measures of Treatment Effect

Given the expected heterogeneity between studies due to our broad focus on all types of MHO's for drug use (e.g., differences in types of MHO's, drugs of use, population), a descriptive synthesis will be conducted instead of meta‐analysis. If primary studies provide continuous outcomes in means/SDs or means/SEs, we will use them to calculate Cohen's *d* and if they report binary outcomes using frequency or proportions, we will use them to calculate odds ratios. These effect sizes will be calculated using the formulas provided in Lipsey and Wilson's 2001 guide. Other formats of reporting will be extracted from included studies and reported in the metric used by the primary studies.

We will report these data using the SWIM guidelines (Campbell et al. 2020). As suggested in the Cochrane Handbook (12.2 and 12.3) we will use tables/figures to visualize and present the information and use vote counting based on the direction of effect to synthesize the data.

#### 3.3.6 Unit of Analysis Issues

Before our descriptive synthesis, we will determine the unit of allocation and, in the case of cluster randomization, will assess and report if the authors have controlled for clustering.

There is no specific duration of follow‐up required for eligibility and it is thus possible that studies will report a variety of follow‐up timepoints. During data synthesis, we plan to group studies that have similar follow‐up timepoints (described in further detail below).

#### 3.3.7 Criteria for Determination of Independent Findings

Before data extraction, reports will be reviewed to determine if there is any overlap in study samples. Reports using the same sample will be linked to ensure that only unique samples are used for analysis.

To ensure independence and an appropriate combination of outcome constructs when descriptively synthesizing the results, outcomes will be split into categories based on similar follow‐up durations (e.g., less than 6 months, 6 months or greater, or 1 year or more) and reported in those categories.

#### 3.3.8 Dealing With Missing Data

We will contact primary study authors to gather any missing data that is necessary to accurately report the primary outcomes of an included study. Other missing data (or if authors do not respond to the queries regarding primary outcomes) will be reported as missing in the review.

#### 3.3.9 Assessment of Heterogeneity

As we do not plan to conduct a meta‐analysis, heterogeneity will be descriptively examined along anticipated areas of difference leading to heterogeneity (study design, risk of bias, MHO, drug of use, population characteristics).

#### 3.3.10 Assessment of Reporting Biases

As we do not plan to conduct a meta‐analysis, we will not have a formal examination of publication bias. We will examine and report the proportion of published versus nonpublished research from our search strategy and descriptively examine whether included unpublished research was more likely to have null or negative findings.

#### 3.3.11 Data Synthesis

Given the expected diversity of populations and settings, we will descriptively synthesize each unique primary outcome across all MHOs, along expected timepoints (< 6 months, 6–12 months, 12+ months) and also for specific MHOs. Individuals often report attending more than one type of MHO in the same period and some studies aggregate attendance across all 12‐step groups or mutual‐help more generally (e.g., Weiss et al. 2005) while others examine effects of attendance at one or two specific MHOs like NA/AA (Gossop et al. 2008) or NA alone (e.g., Christo 1995). Thus, we will attempt to examine differences by type of MHO attended when possible (i.e., when reported by primary studies), but anticipate that some analyses will only allow for an examination of any type of MHO attendance.

The analysis will also synthesize TSF Studies separately from studies examining the sole influence of MHO participation on outcomes.

#### 3.3.12 Subgroup Analysis and Investigation of Heterogeneity

As we do not plan to conduct a meta‐analysis, heterogeneity will be descriptively examined along anticipated areas of difference leading to heterogeneity (study design, risk of bias, MHO, primary drug of use, population characteristics, etc.).

#### 3.3.13 Sensitivity Analysis

Not applicable.

#### 3.3.14 Treatment of Qualitative Research

We do not plan to include qualitative research.

#### 3.3.15 Summary of Findings and Assessment of the Certainty of the Evidence

We do not plan to include the GRADE summary of findings and assessment of the certainty of the evidence tables in this review given resource limitations.

## Author Contributions

Content: John F. Kelly, Brandon G. Bergman, Emily A. Hennessy. Systematic review methods: Emily A. Hennessy, Jenny O'Connor, Morgan Klein. Statistical analysis: Emily A. Hennessy. Information retrieval: Leonard L. Levin.

## Conflicts of Interest

John F. Kelly has published a prior Cochrane Collaboration review on mutual‐help for AUD and has otherwise published numerous reviews and empirical reports in this area. He has also received funding from the US National Institutes of Health, SAMHSA, and private foundations, to conduct research in this area. John will not be part of the coding for any of the included studies.

Emily A. Hennessy is co‐chair of the SUTR group, so the protocol and review would need to be managed by Noel Vest (the other Co‐Chair). Brandon G. Bergman is a content expert of the SUTR group, so would not be part of the Campbell review of protocol and final review process.

## Preliminary Timeframe

- Date you plan to submit a draft protocol: August 15, 2024.
- Date you plan to submit a draft review: June 30, 2025.

## Plans for Updating This Review

We plan to update the review in approximately 5 years, pending funding.

## Data and Analytic Code

Metadata and data will be made available on a publicly available OSF as part of the final publication.

## Sources of Support

### Internal Sources

This work was funded in part by the Massachusetts General Hospital Recovery Research Institute.

### 1 External Sources

This work was funded in part by the U.S. Substance Abuse and Mental Health Administration's Opioid Response Network (1H79TI085588).

## Supporting information

Supporting information.

Supporting information.
