# Supplementary material for: PROTOCOL: Mutual Help Organizations to Support Recovery Among Individuals Who Use Drugs: A Systematic Review Protocol
Source: Campbell Syst Rev. 2025 Mar 4;21(1):e70021. doi: 10.1002/cl2.70021 (PMC11876998; doi:10.1002/cl2.70021)
Supplement: Supplementary file 1 — Supporting information. [file CL2-21-e70021-s002.pdf]

Data Extraction

NOTE: THIS IS A PDF OF REDCAP DATA EXTRACTION PAGES. THE FORMATTING AND BRANCHING LOGIC WILL NOT DISPLAY PROPERLY.

Coder initials (First, Middle, Last)

____
(Enter in all CAPS)

Note: For any missing information, enter -999 as a response in the coding form.

Enter the Covidence article ID number

____

Study Citation (authors and year)

____

Is reference harvesting completed?

- ◯ Yes
- ◯ No

Is this study connected to other studies in the dataset (i.e., a manuscript from the same sample as another study)?

- ◯ Yes
- ◯ No

Enter the report numbers (REDCap assigned record ID) of the other studies, separated by a colon. If this is the first instance of the overall study being reported, leave this field blank.

____

Describe the study aims using the author's words, include the page number
Note: Likely found in abstract or in sentences immediately prior to the methods.

____

Study Design

- ◯ RCT
- ◯ QED
- ◯ Other

What type of study was this?

- ◯ Cross-sectional
- ◯ Longitudinal

What were the mutual help organizations of focus? Check all that apply.

- ☐ Celebrate Recovery
- ☐ Cocaine Anonymous (CA)
- ☐ Crystal Methamphetamine Anonymous (CMA)
- ☐ Methadone Anonymous
- ☐ LifeRing Secular Recovery
- ☐ Marijuana Anonymous (MA)
- ☐ Narcotics Anonymous (NA)
- ☐ Refuge Recovery/Recovery Dharma
- ☐ Secular Organizations for Sobriety (S.O.S.)
- ☐ SMART Recovery
- ☐ Women for Sobriety
- ☐ Any (not specified)
- ☐ Other

List other mutual help organizations:

____

How many groups were used in this study?	◯ 2<br>◯ 3<br>◯ 4<br>◯ 5
How many groups were of the primary intervention, an MHO for drug use?<br><br>Example:<br><br>You would code 2 here if the study had two conditions and each condition was a different eligible MHO (for example, Narcotics Anonymous was Group 1 and SMART was Group 2).<br><br>You would code 1 here if the study had two condition and one condition was an eligible MHO, but the other condition was a non-MHO attending group.	◯ 1<br>◯ 2<br>◯ 3<br>◯ 4
How many groups were of a control or comparator condition that were not examining an MHO for drug use?<br><br>Example:<br><br>You would code 0 here if the study had two conditions and both conditions were focused on unique, eligible MHOs.<br><br>You would code 1 here if the study had two conditions and one condition was an eligible MHO, but the other condition was a non-MHO attending group.<br><br>You would code 2 here if the study had three conditions and one condition was an eligible MOH but each comparator was on a different condition (e.g., non-support seeking, alternative support).	◯ 0<br>◯ 1<br>◯ 2<br>◯ 3<br>◯ 4
Name the primary MHO intervention (or first alphabetically if more than 1 included in this study)	__________________________________
Describe the primary MHO intervention (or first alphabetically if more than 1 included in this study)	____
Name the second MHO intervention	__________________________________
Describe the second MHO intervention	____
Name the third MHO intervention	__________________________________
Describe the third MHO intervention	____

Name the control or comparator(s) ________________

Describe the control or comparator(s) ________________

Name the second control/comparator condition ________________

Describe the second control/comparator condition ________________

Name the third control/comparator condition ________________

Describe the third control/comparator condition ________________

Describe the overall sample ________________

Total Sample Size (recruited) ________________

Total Analytic Sample Size. ________________

Note: This will only be coded if the authors indicated that cases were dropped prior to analysis for the entire datset - the sample size for each outcome will be coded in the outcomes field as this may differ due to attrition.

Total Sample Characteristics

Enter the mean age of the sample ________________
(Enter -999 if not reported)

Enter the SD of the age of the sample ________________
(Enter -999 if not reported)

Enter the lower end of the age range of the sample ________________
(Enter -999 if not reported)

Enter the upper end of the age range of the sample ________________
(Enter -999 if not reported)

What percent of the sample was female? ________________
(Enter as full percent value: type 90% vs 0.90)

What percent of the sample was male?

______________________________ (Enter as full percent value: type 90% vs 0.90)

What percent of the sample was White?

______________________________ (Enter as full percent value: type 90% vs 0.90)

What percent of the sample was Black?

______________________________ (Enter as full percent value: type 90% vs 0.90)

What percent of the sample was Asian?

______________________________ (Enter as full percent value: type 90% vs 0.90)

What percent of the sample was Native American?

______________________________ (Enter as full percent value: type 90% vs 0.90)

What percent of the sample was Hispanic?

______________________________ (Enter as full percent value: type 90% vs 0.90)

What percent of the sample was "other" race/ethnicity not listed above (or combination of the above if the authors do not split it out)?

______________________________ (Enter as full percent value: type 90% vs 0.90)

Intervention/Treatment/Experimental Condition Characteristics

What was the sample size of the experimental/treatment/intervention condition?

____

Enter the mean age of the treatment/intervention/experimental group

______________________________ (Enter -999 if not reported)

Enter the SD of the age of the treatment/intervention/experimental group

______________________________ (Enter -999 if not reported)

What percent of the treatment/intervention/experimental group was female?

______________________________ (Enter as full percent value: type 90% vs 0.90)

What percent of the treatment/intervention/experimental group was male?

______________________________ (Enter as full percent value: type 90% vs 0.90)

What percent of the treatment/intervention/experimental group was White?

______________________________ (Enter as full percent value: type 90% vs 0.90)

What percent of the treatment/intervention/experimental group was Black?

______________________________ (Enter as full percent value: type 90% vs 0.90)

What percent of the treatment/intervention/experimental group was Asian?

____
(Enter as full percent value: type 90% vs 0.90)

What percent of the treatment/intervention/experimental group was Hispanic?

____
(Enter as full percent value: type 90% vs 0.90)

What percent of the treatment/intervention/experimental group was "other" race/ethnicity not listed above (or combination of the above if the authors do not split it out)?

____
(Enter as full percent value: type 90% vs 0.90)

Second Intervention/Treatment/Experimental Condition Characteristics

What was the sample size of the second experimental/treatment/intervention condition?

____

Enter the mean age of the second treatment/intervention/experimental group

____
(Enter -999 if not reported)

Enter the SD of the age of the second treatment/intervention/experimental group

____
(Enter -999 if not reported)

What percent of the second treatment/intervention/experimental group was female?

____
(Enter as full percent value: type 90% vs 0.90)

What percent of the second treatment/intervention/experimental group was male?

____
(Enter as full percent value: type 90% vs 0.90)

What percent of the second treatment/intervention/experimental group was White?

____
(Enter as full percent value: type 90% vs 0.90)

What percent of the second treatment/intervention/experimental group was Black?

____
(Enter as full percent value: type 90% vs 0.90)

What percent of the second treatment/intervention/experimental group was Asian?

____
(Enter as full percent value: type 90% vs 0.90)

What percent of the second treatment/intervention/experimental group was Hispanic?

____
(Enter as full percent value: type 90% vs 0.90)

What percent of the second treatment/intervention/experimental group was "other" race/ethnicity not listed above (or combination of the above if the authors do not split it out)?

____
(Enter as full percent value: type 90% vs 0.90)

Control/Comparator Group Characteristics

What was the sample size of the control/comparison condition? ______________________

Enter the mean age of the control/comparator group ______________________
(Enter -999 if not reported)

Enter the SD of the age of the control/comparator group ______________________
(Enter -999 if not reported)

What percent of the control/comparator group was female? ______________________
(Enter as full percent value: type 90% vs 0.90)

What percent of the control/comparator group was male? ______________________
(Enter as full percent value: type 90% vs 0.90)

What percent of the control/comparator group was White? ______________________
(Enter as full percent value: type 90% vs 0.90)

What percent of the control/comparator group was Black? ______________________
(Enter as full percent value: type 90% vs 0.90)

What percent of the control/comparator group was Asian? ______________________
(Enter as full percent value: type 90% vs 0.90)

What percent of the control/comparator group was Hispanic? ______________________
(Enter as full percent value: type 90% vs 0.90)

What percent of the control/comparator group was "other" race/ethnicity not listed above (or combination of the above if the authors do not split it out)? ______________________
(Enter as full percent value: type 90% vs 0.90)

Second Control/Comparator Group Characteristics

What was the sample size of the second control/comparison condition? ______________________

Enter the mean age of the second control/comparator group ______________________
(Enter -999 if not reported)

Enter the SD of the age of the second control/comparator group ______________________
(Enter -999 if not reported)

What percent of the second control/comparator group was female?

____
(Enter as full percent value: type 90% vs 0.90)

What percent of the second control/comparator group was male?

____
(Enter as full percent value: type 90% vs 0.90)

What percent of the second control/comparator group was White?

____
(Enter as full percent value: type 90% vs 0.90)

What percent of the second control/comparator group was Black?

____
(Enter as full percent value: type 90% vs 0.90)

What percent of the second control/comparator group was Asian?

____
(Enter as full percent value: type 90% vs 0.90)

What percent of the second control/comparator group was Hispanic?

____
(Enter as full percent value: type 90% vs 0.90)

What percent of the second control/comparator group was "other" race/ethnicity not listed above (or combination of the above if the authors do not split it out)?

____
(Enter as full percent value: type 90% vs 0.90)

Other Study Characteristics

How many study follow-up timepoints (outcome assessments) were used?
Note: If this is an EMA-style study assessment, speak to the study team about how to count assessments

- ◯ 1
- ◯ 2
- ◯ 3
- ◯ 4
- ◯ 5
- ◯ 6
- ◯ 7
- ◯ 8
- ◯ 9
- ◯ 10

(DO NOT INCLUDE the baseline assessment)

Enter the length of time for the first study follow-up (in weeks)

____
(Multiply months by 4.3)

What was the retention rate for the first study follow-up?

____
(Enter as percent, if possible)

Enter the length of time for the second study follow-up (in weeks)

____
(Multiply months by 4.3)

What was the retention rate for the second study follow-up?

____
(Enter as percent, if possible)

Enter the length of time for the third study follow-up (in weeks)

____
(Multiply months by 4.3)

What was the retention rate for the third study follow-up?

____
(Enter as percent, if possible)

Enter the length of time for the fourth study follow-up (in weeks)

____
(Multiply months by 4.3)

What was the retention rate for the fourth study follow-up?

____
(Enter as percent, if possible)

Enter the length of time for the fifth study follow-up (in weeks)

____
(Multiply months by 4.3)

What was the retention rate for the fifth study follow-up?

____
(Enter as percent, if possible)

Enter the length of time for the sixth study follow-up (in weeks)

____
(Multiply months by 4.3)

What was the retention rate for the sixth study follow-up?

____
(Enter as percent, if possible)

What was the overall retention rate?

____
(Enter as percent, if possible)

What were the primary substances or addictions of focus? Check all that apply.

- ☐ Cannabis/Marijuana
- ☐ Crack cocaine
- ☐ Cocaine
- ☐ Heroin
- ☐ Methamphetamine
- ☐ Opioids
- ☐ Stimulants
- ☐ Any substance (not specified but NOT alcohol)
- ☐ Multi-substance
- ☐ Other
- ☐ Unclear/Not reported

Describe the primary substances or addictions of focus in this study. Included relevant Ns/% if the sample is described as having multiple substance use issues.

____

Select which primary and secondary outcomes you will be coding for this study.

PRIMARY (SUBSTANCE USE) Check circle if coding this outcome # of Timepoints to Code # of outcome types by timepoints (multiply)

Drug use frequency Alcohol:

______

Cocaine:

______

Heroin:

______

Marijuana:

______

Opiates: ______

Stimulants: ______

Other specific drug: ______

Any (unspecified):______

Mixed: ______

Sum of all substances: ______

______ ______

Drug use quantity Alcohol:

______

Cocaine:

______

Heroin:

______

Marijuana:

______

Opiates: ______

Stimulants: ______

Other specific drug: ______

Any (unspecified):______

Mixed: ______

Sum of all substances: ______

______ ______

Abstinence/non-abstinence Alcohol:

______

Cocaine:

______

Heroin:

______

Marijuana:

______

Opiates: ______

Stimulants: ______

Other specific drug: ______

Any (unspecified):______

Mixed: ______

Sum of all substances: ______

______ ______

Addiction severity (e.g., LDQ score) Alcohol:

______

Cocaine:

______

Heroin:

______

Marijuana:

______

Opiates: ______

Stimulants: ______

Other specific drug: ______

Any (unspecified):______

Mixed: ______

Sum of all substances: ______

______ ______

SECONDARY Check circle if coding this outcome Indicate how many different types of each outcome to code # of Timepoints to Code # of outcome types by timepoints (multiply)

Cost-benefit/cost-effectiveness ______ ______ ______ ______

Crime/Criminal involvement ______ ______ ______ ______

Employment/Training

______ ______ ______ ______

Healthcare utilization ______ ______ ______ ______

Housing

______ ______ ______ ______

Mental or Physical Health ______ ______ ______ ______

Recovery Capital

______ ______ ______ ______

Social network change ______ ______ ______ ______

Well-being/Quality of life ______ ______ ______ ______

Other negative consequences due to drug use ______ ______ ______ ______

SUMMARY ______ ______ ______ ______

Enter any notes or questions encountered during coding here.

____

Quality Assessment

Dictionary of terms and ratings guidance for each section

[Attachment: "qualilty-assessment-dictionary_2010bb4cc44d23b4446e95ef53f3430f51c6.pdf"]

Selection Bias

Are the individuals selected to participate in the study likely to be representative of the target population?	◯ Very likely<br>◯ Somewhat likely<br>◯ Not likely<br>◯ Can't tell
What percentage of selected individuals agreed to participate?	◯ 80 - 100% agreement<br>◯ 60 - 79% agreement<br>◯ less than 60% agreement<br>◯ Not applicable<br>◯ Can't tell
Rate the section on selection bias.	◯ Strong<br>◯ Moderate<br>◯ Weak

Study Design

Was the study described as randomized?	◯ Yes<br>◯ No
Was the method of randomization described?	◯ Yes<br>◯ No
Was the method (of randomization) appropriate?	◯ Yes<br>◯ No
Rate the section on study design.	◯ Strong<br>◯ Moderate<br>◯ Weak

Confounders

Were there important differences between groups prior to the intervention?	◯ Yes<br>◯ No<br>◯ Cannot tell
Select which of the following typical confounders were present in this study and were controlled (either in the design (e.g. stratification, matching) or analysis):	☐ Race<br>☐ Sex<br>☐ Marital status/family<br>☐ Age<br>☐ SES (income or class)<br>☐ Education<br>☐ Health status<br>☐ Pre-intervention score on outcome measure

Indicate the percentage of relevant confounders that were controlled (either in the design (e.g. stratification, matching) or analysis)?	◯ 80 - 100% (most)<br>◯ 60 - 79% (some)<br>◯ Less than 60% (few or none)<br>◯ Can't Tell
Rate the section on confounders.	◯ Strong<br>◯ Moderate<br>◯ Weak

Blinding

Was (were) the outcome assessor(s) aware of the intervention or exposure status of participants?	◯ No<br>◯ Yes<br>◯ Can't tell
Were the study participants aware of the research question?	◯ No<br>◯ Yes<br>◯ Can't tell
Rate the section on blinding.	◯ Strong<br>◯ Moderate<br>◯ Weak<br>◯ Not Applicable

Data Collection Methods

Were data collection tools shown to be valid?	◯ No<br>◯ Yes<br>◯ Can't tell
Were data collection tools shown to be reliable?	◯ No<br>◯ Yes<br>◯ Can't tell
Rate the section on data collection methods.	◯ Strong<br>◯ Moderate<br>◯ Weak

Withdrawals and dropouts

Were withdrawals and drop-outs reported in terms of numbers and/or reasons per group?	◯ No<br>◯ Yes<br>◯ Can't tell<br>◯ Not Applicable (i.e. one time surveys or interviews)
Indicate the percentage of participants completing the study. (If the percentage differs by groups, record the lowest).	◯ 80 -100%<br>◯ 60 - 79%<br>◯ less than 60%<br>◯ Can't tell<br>◯ Not Applicable (i.e. Retrospective case-control)
Rate the section on withdrawals and dropouts.	◯ Strong<br>◯ Moderate<br>◯ Weak<br>◯ Not Applicable

Intervention Integrity

What percentage of participants received the allocated intervention or exposure of interest?

- ◯ 80 -100%
- ◯ 60 - 79%
- ◯ less than 60%
- ◯ Can't tell

Was the consistency of the intervention measured?

- ◯ No
- ◯ Yes
- ◯ Can't tell

Is it likely that subjects received an unintended intervention (contamination or co-intervention) that may influence the results?

- ◯ No
- ◯ Yes
- ◯ Can't tell

Analyses

Indicate the unit of allocation (check one)

- ◯ Community
- ◯ Organization/Institution
- ◯ Practice/Office
- ◯ Individual
- ◯ Not applicable

Indicate the unit of analysis (check one)

- ◯ Community
- ◯ Organization/Institution
- ◯ Practice/Office
- ◯ Individual
- ◯ Not applicable

Are the statistical methods appropriate for the study design?

- ◯ No
- ◯ Yes
- ◯ Can't tell

Is the analysis performed by intervention allocation status (i.e. intention to treat) rather than the actual intervention received?

- ◯ No
- ◯ Yes
- ◯ Can't tell

OVERALL RATINGS

SUMMARY OF QUALITY RATINGS
Selection Bias [rate_a]
Study Design [rate_b]
Confounders [rate_c]
Blinding [rate_d]
Data Collection Method [rate_e]
Withdrawals and dropouts [rate_f]

Global rating for this paper:

- ◯ Strong (no WEAK ratings)
- ◯ Moderate (one WEAK rating)
- ◯ Weak (two or more WEAK ratings)

Global rating for this paper - use for robvis tool.
SIMPLY RE-ENTER YOUR ORIGINAL GLOBAL RATING

(renamed and recoded for simplified upload)

1, Strong (no WEAK ratings)
2, Moderate (one WEAK rating)
3, Weak (two or more WEAK ratings)

◯ Strong
◯ Moderate
◯ Weak

Outcomes - Substance Use (Frequency)

NOTE: THIS IS AN EXAMPLE FOR ONE OUTCOME TYPE.
THE PAGE WILL BE REPLICATED FOR ALL OUTCOMES

Record ID ______________________

If there is only one outcome measure for this study, you will create only one record in this worksheet. If there are three outcomes, you will create three instances.

If there are longitudinal outcomes, code the outcomes in order consecutively, starting with baseline and ending with the longest timepoint.

If there are multiple types of outcomes that fit within this coding category and they are longitudinal, code a single type of outcome from baseline to endpoint (in order consecutively) and then code the next single type baseline to endpoint, until all outcomes are coded.

Based on your entry on the first coding sheet for this study, you will be coding [outcomet_duf] outcomes for [multiplegrn] groups.

Specific type of substance use

- ◯ Alcohol: Any measure of alcohol use
- ◯ Cocaine: Any measure of cocaine use
- ◯ Heroin: Any measure of heroin use
- ◯ Marijuana: Any measure of marijuana use
- ◯ Opiate: Any measure of opiate use
- ◯ Stimulant: Any measure of stimulant use
- ◯ Other Specific Substance: Any measure of use of drugs other than those listed above; can be use of one drug or use of a combination of drugs that does not include alcohol or marijuana
- ◯ Mixed Substance Use: Any measure of use of a substance that includes more than one substance

Write the label the authors give the outcome verbatim from the text. ______________________

What was the timeframe for this outcome? Note, indicate in months. For baseline, use "0".

For each outcome timepoint, create a new outcome record.

____
(Numeric responses only)

Who provided the original information for this outcome?

- ◯ Self-report (including participant filling out survey)
- ◯ Other report
- ◯ Official records (e.g., court, school)
- ◯ Physiological (e.g., urine testing)
- ◯ Multiple sources, cannot tell which is dominant
- ◯ Cannot tell

Describe the way the outcome was measured and write in a brief description of the outcome you are coding.<br><br>This should include the authors' label for this variable (e.g., average number of alcoholic beverages consumed each week), the instrument (e.g., Timeline Followback), and information about what is being measured (e.g., level of consumption of alcohol, etc.). Quote or closely paraphrase the description that is provided in the original report. You will likely find this information in the methods section of the article.	________________________________
Indicate the direction of scoring.<br><br>For example, if they had higher recovery capital scores, they did better. If they had greater return to use, they did worse.	◯ Higher scores: someone did BETTER<br>◯ Higher scores: someone did WORSE
I have the following statistical information about this outcome (select one)	◯ Means and SD (Standard Deviations) for each group<br>◯ Means and SE (Standard Errors) for each group<br>◯ Frequency of event/occurrence for each group<br>◯ Percent of group with event<br>◯ Some other statistical information<br>◯ Narrative results

Provide the following information about this outcome for each group where there is information on this outcome:

Groups	N	Mean	Standard Deviation	Standard Error	Percent with event	N with Event	N without event
Select treatment 1:	______	______	______	______	______	______	______
Select treatment 2:	______	______	______	______	______	______	______
Select treatment 3:	______	______	______	______	______	______	______
Select control/comparator 1:	______	______	______	______	______	______	______
Select control/comparator 2:	______	______	______	______	______	______	______

Describe the findings of the analysis for this outcome. Include as much statistical detail as is provided by the authors.	________________________________
