# Supplementary material for: PROTOCOL: Mutual Help Organizations to Support Recovery Among Individuals Who Use Drugs: A Systematic Review Protocol
Source: Campbell Syst Rev. 2025 Mar 4;21(1):e70021. doi: 10.1002/cl2.70021 (PMC11876998; doi:10.1002/cl2.70021)
Supplement: Supplementary file 2 — Supporting information. [file CL2-21-e70021-s001.docx]

# Appendices

##### Appendices should be submitted as [supplementary material](https://authorservices.wiley.com/author-resources/Journal-Authors/Prepare/manuscript-preparation-guidelines.html/supporting-information.html).

## Search strategies:

**(1) Medline (OVID)**

1. "Substance-Related Disorders"/ OR "Amphetamine-Related Disorders"/ OR "Cocaine-Related Disorders"/ OR exp "Drug Misuse"/ OR "inhalant abuse"/ OR "Marijuana abuse"/ OR exp "Narcotic-related disorders"/ OR "phencyclidine abuse"/ OR "Substance Abuse, Intravenous"/ OR "Substance Abuse, oral"/ OR "substance withdrawal syndrome"/ OR exp "Substance-Related Disorders"/rh OR (((cannabis OR cocaine OR crack OR drug? OR substance? OR hallucinogen* OR hash* OR heroin OR inhalant? OR methamphetamine* OR psilocybin OR LSD OR meth OR marijuana OR multidrug OR phencyclidine OR polydrug OR polysubstance) adj1 ("use" OR user? OR using OR used)) OR ((drug? OR multidrug OR polydrug OR polysubstance OR substance? OR amphetamine* OR cannabis OR crack OR cocaine OR hallucinogen* OR hash* OR heroin OR inhalant? OR methamphetamine* OR meth OR marijuana OR phencyclidine OR narcotic* OR opioid? OR opiate? OR sedative? OR stimulant? OR psilocybin OR psychedelic? OR LSD) adj2 (abuse* OR abusing OR addict* OR depend* OR misuse? OR disorder? OR overuse? OR habit* OR rehab* OR recovery OR detox*)) OR chemical*-depend*).ti,ab,kf,kw.

2. Self-Help Groups/ OR (((narcotics OR meth OR methamphetamine* OR cannabis OR marijuana OR cocaine OR methadone OR suboxone) adj2 (anonymous OR self-help OR support-group* OR peer-support* OR group-therap* OR therap*-group? OR recovery-communit*)) OR ((community-support* OR support*-community OR peer-support* OR self-help) adj1 (recovery OR clean-living OR sober OR sobriety OR drug-free OR substance-free OR detox*)) OR virtual-NA OR NA-meeting? OR 12-step? OR twelve-step? OR "celebrate recovery" OR LifeRing OR "refuge recovery" OR "recovery dharma" OR "secular recovery" OR "secular sobriety" OR secular-organization*-for-sobriety OR "SMART recovery" OR "community recovery" OR mutual-aid OR mutual-help* OR "self-management in recovery" OR mutual-support* OR (("web" OR online) adj1 forum*)).ti,ab,kf,kw.

3. 1 and 2

**(2) Embase (Elsevier)**

('drug dependence'/exp NOT 'alcoholism' OR ((cannabis OR cocaine OR crack OR drug? OR substance? OR hallucinogen* OR hash* OR heroin OR inhalant? OR methamphetamine* OR psilocybin OR lsd OR meth OR marijuana OR multidrug OR phencyclidine OR polydrug OR polysubstance) NEXT/2 ('use' OR user? OR using OR used)) OR ((drug? OR multidrug OR polydrug OR polysubstance OR substance? OR amphetamine* OR cannabis OR crack OR cocaine OR hallucinogen* OR hash* OR heroin OR inhalant? OR methamphetamine* OR meth OR marijuana OR phencyclidine OR narcotic* OR opioid? OR opiate? OR sedative? OR stimulant? OR psilocybin OR psychedelic? OR lsd) NEXT/2 (abuse* OR abusing OR addict* OR depend* OR misuse? OR disorder? OR overuse? OR habit* OR rehab* OR recovery OR detox*)) OR 'chemical* depend*') AND ('self help'/exp OR 'twelve step facilitation'/exp OR ((narcotics OR meth OR methamphetamine* OR cannabis OR marijuana OR cocaine OR methadone OR suboxone) NEXT/2 (anonymous OR 'self help' OR 'support group*' OR 'peer support*' OR 'group therap*' OR 'therap* group?' OR 'recovery communit*')) OR (('community support*' OR 'support* community' OR 'peer support*' OR 'self help') NEXT/2 (recovery OR 'clean living' OR sober OR sobriety OR 'drug free' OR 'substance free' OR detox*)) OR 'virtual na' OR 'na meeting?' OR '12 step?' OR 'twelve step?' OR 'celebrate recovery' OR lifering OR 'refuge recovery' OR 'recovery dharma' OR 'secular recovery' OR 'secular sobriety' OR 'secular organization* for sobriety' OR 'smart recovery' OR 'community recovery' OR 'mutual aid' OR 'mutual help*' OR 'self-management in recovery' OR 'mutual support*' OR (('web' OR online) AND adj1 AND forum*))

**(3) PsycInfo (OVID)**

| 1 | exp "Substance Use Disorder"/ or exp Drug Addiction/ or exp Drug Abuse/ or exp Drug Dependency/ |
| --- | --- |
| 2 | (((cannabis or cocaine or crack or drug? or substance? or hallucinogen* or hash* or heroin or inhalant? or methamphetamine* or psilocybin or LSD or meth or marijuana or multidrug or phencyclidine or polydrug or polysubstance) adj1 ("use" or user? or using or used)) or ((drug? or multidrug or polydrug or polysubstance or substance? or amphetamine* or cannabis or crack or cocaine or hallucinogen* or hash* or heroin or inhalant? or methamphetamine* or meth or marijuana or phencyclidine or narcotic* or opioid? or opiate? or sedative? or stimulant? or psilocybin or psychedelic? or LSD) adj2 (abuse* or abusing or addict* or depend* or misuse? or disorder? or overuse? or habit* or rehab* or recovery or detox*)) or chemical*-depend*).ti,ab. |

3 exp Support Groups/ or exp Twelve Step Programs/

4 (((narcotics or meth or methamphetamine* or cannabis or marijuana or cocaine or methadone or suboxone) adj2 (anonymous or self-help or support-group* or peer-support* or group-therap* or therap*-group? or recovery-communit*)) or ((community-support* or support*-community or peer-support* or self-help) adj1 (recovery or clean-living or sober or sobriety or drug-free or substance-free or detox*)) or virtual-NA or NA-meeting? or 12-step? or twelve-step? or "celebrate recovery" or LifeRing or "refuge recovery" or "recovery dharma" or "secular recovery" or "secular sobriety" or secular-organization*-for-sobriety or "SMART recovery" or "community recovery" or mutual-aid or mutual-help* or "self-management in recovery" or mutual-support* or (("web" or online) adj1 forum*)).ti,ab.

| 5 | 1 or 2 |
| --- | --- |
| 6 | 3 or 4 |
| 7 | 5 and 6 |
| 8 | alcoholism/ or alcohols/ |
| 9 | 7 not 8 |

**(4) CINAHL (EBSCO)**

| S14 | S10 AND S13 | Expanders - Apply equivalent subjects  Search modes - Proximity | [View Results](javascript:__doPostBack('ctl00$ctl00$FindField$FindField$historyControl$HistoryRepeater$ctl00$linkResults','')) (1,265)  [View Details](javascript:showShDetails(%22ctl00_ctl00_FindField_FindField_historyControl_ctrlPopup%22,%20%22S14%22,%20true);)  [Edit](https://web-p-ebscohost-com.ezp-prod1.hul.harvard.edu/Legacy/Views/UserControls/EHOST/) |  |
| --- | --- | --- | --- | --- |
|  | S13 | S11 OR S12 | Expanders - Apply equivalent subjects  Search modes - Proximity | [View Results](javascript:__doPostBack('ctl00$ctl00$FindField$FindField$historyControl$HistoryRepeater$ctl01$linkResults','')) (14,247)  [View Details](javascript:showShDetails(%22ctl00_ctl00_FindField_FindField_historyControl_ctrlPopup%22,%20%22S13%22,%20true);)  [Edit](https://web-p-ebscohost-com.ezp-prod1.hul.harvard.edu/Legacy/Views/UserControls/EHOST/) |
|  | S12 | (((narcotics OR meth OR methamphetamine* OR cannabis OR marijuana OR cocaine OR methadone OR suboxone) Nx (anonymous OR self-help OR support-group* OR peer-support* OR group-therap* OR therap*-group* OR recovery-communit*)) OR ((community-support* OR support*-community OR peer-support* OR self-help) Nx (recovery OR clean-living OR sober OR sobriety OR drug-free OR substance-free OR detox*)) OR virtual-NA OR NA-meeting* OR 12-step* OR twelve-step* OR "celebrate recovery" OR LifeRing OR "refuge re [...](javascript:showHistoryTerm('ctl00_ctl00_FindField_FindField_historyControl_HistoryRepeater_ctl02_ellipsis',true)) | Expanders - Apply equivalent subjects  Search modes - Proximity | [View Results](javascript:__doPostBack('ctl00$ctl00$FindField$FindField$historyControl$HistoryRepeater$ctl02$linkResults','')) (2,357)  [View Details](javascript:showShDetails(%22ctl00_ctl00_FindField_FindField_historyControl_ctrlPopup%22,%20%22S12%22,%20true);)  [Edit](https://web-p-ebscohost-com.ezp-prod1.hul.harvard.edu/Legacy/Views/UserControls/EHOST/) |
|  | S11 | (MH "Support Groups+") OR ("twelve step program*") | Expanders - Apply equivalent subjects  Search modes - Proximity | [View Results](javascript:__doPostBack('ctl00$ctl00$FindField$FindField$historyControl$HistoryRepeater$ctl03$linkResults','')) (12,545)  [View Details](javascript:showShDetails(%22ctl00_ctl00_FindField_FindField_historyControl_ctrlPopup%22,%20%22S11%22,%20true);)  [Edit](https://web-p-ebscohost-com.ezp-prod1.hul.harvard.edu/Legacy/Views/UserControls/EHOST/) |
|  | S10 | S8 OR S9 | Expanders - Apply equivalent subjects  Search modes - Proximity | [View Results](javascript:__doPostBack('ctl00$ctl00$FindField$FindField$historyControl$HistoryRepeater$ctl04$linkResults','')) (113,900)  [View Details](javascript:showShDetails(%22ctl00_ctl00_FindField_FindField_historyControl_ctrlPopup%22,%20%22S10%22,%20true);)  [Edit](https://web-p-ebscohost-com.ezp-prod1.hul.harvard.edu/Legacy/Views/UserControls/EHOST/) |
|  | S9 | (((cannabis OR cocaine OR crack OR drug* OR substance* OR hallucinogen* OR hash* OR heroin OR inhalant* OR methamphetamine* OR psilocybin OR LSD OR meth OR marijuana OR multidrug OR phencyclidine OR polydrug OR polysubstance) Nx ("use" OR user* OR using OR used)) OR ((drug* OR multidrug OR polydrug OR polysubstance OR substance* OR amphetamine* OR cannabis OR crack OR cocaine OR hallucinogen* OR hash* OR heroin OR inhalant* OR methamphetamine* OR meth OR marijuana OR phencyclidine OR narcotic*  [...](javascript:showHistoryTerm('ctl00_ctl00_FindField_FindField_historyControl_HistoryRepeater_ctl05_ellipsis',true)) | Expanders - Apply equivalent subjects  Search modes - Proximity | [View Results](javascript:__doPostBack('ctl00$ctl00$FindField$FindField$historyControl$HistoryRepeater$ctl05$linkResults','')) (777)  [View Details](javascript:showShDetails(%22ctl00_ctl00_FindField_FindField_historyControl_ctrlPopup%22,%20%22S9%22,%20true);)  [Edit](https://web-p-ebscohost-com.ezp-prod1.hul.harvard.edu/Legacy/Views/UserControls/EHOST/) |
|  | S8 | S1 OR S2 OR S3 OR S4 OR S5 OR S6 OR S7 | Expanders - Apply equivalent subjects  Search modes - Proximity | [View Results](javascript:__doPostBack('ctl00$ctl00$FindField$FindField$historyControl$HistoryRepeater$ctl06$linkResults','')) (113,658)  [View Details](javascript:showShDetails(%22ctl00_ctl00_FindField_FindField_historyControl_ctrlPopup%22,%20%22S8%22,%20true);)  [Edit](https://web-p-ebscohost-com.ezp-prod1.hul.harvard.edu/Legacy/Views/UserControls/EHOST/) |
|  | S7 | (MH "Methamphetamine") | Expanders - Apply equivalent subjects  Search modes - Proximity | [View Results](javascript:__doPostBack('ctl00$ctl00$FindField$FindField$historyControl$HistoryRepeater$ctl07$linkResults','')) (3,127)  [View Details](javascript:showShDetails(%22ctl00_ctl00_FindField_FindField_historyControl_ctrlPopup%22,%20%22S7%22,%20true);)  [Edit](https://web-p-ebscohost-com.ezp-prod1.hul.harvard.edu/Legacy/Views/UserControls/EHOST/) |
|  | S6 | (MH "Hallucinogens") OR (MH "Phencyclidine") | Expanders - Apply equivalent subjects  Search modes - Proximity | [View Results](javascript:__doPostBack('ctl00$ctl00$FindField$FindField$historyControl$HistoryRepeater$ctl08$linkResults','')) (1,647)  [View Details](javascript:showShDetails(%22ctl00_ctl00_FindField_FindField_historyControl_ctrlPopup%22,%20%22S6%22,%20true);)  [Edit](https://web-p-ebscohost-com.ezp-prod1.hul.harvard.edu/Legacy/Views/UserControls/EHOST/) |
|  | S5 | (MH "Narcotics") | Expanders - Apply equivalent subjects  Search modes - Proximity | [View Results](javascript:__doPostBack('ctl00$ctl00$FindField$FindField$historyControl$HistoryRepeater$ctl09$linkResults','')) (13,333)  [View Details](javascript:showShDetails(%22ctl00_ctl00_FindField_FindField_historyControl_ctrlPopup%22,%20%22S5%22,%20true);)  [Edit](https://web-p-ebscohost-com.ezp-prod1.hul.harvard.edu/Legacy/Views/UserControls/EHOST/) |
|  | S4 | (MH "Cocaine") OR (MH "Crack Cocaine") | Expanders - Apply equivalent subjects  Search modes - Proximity | [View Results](javascript:__doPostBack('ctl00$ctl00$FindField$FindField$historyControl$HistoryRepeater$ctl10$linkResults','')) (5,469)  [View Details](javascript:showShDetails(%22ctl00_ctl00_FindField_FindField_historyControl_ctrlPopup%22,%20%22S4%22,%20true);)  [Edit](https://web-p-ebscohost-com.ezp-prod1.hul.harvard.edu/Legacy/Views/UserControls/EHOST/) |
|  | S3 | (MH "Amphetamines") OR (MH "Amphetamine") | Expanders - Apply equivalent subjects  Search modes - Proximity | [View Results](javascript:__doPostBack('ctl00$ctl00$FindField$FindField$historyControl$HistoryRepeater$ctl11$linkResults','')) (2,124)  [View Details](javascript:showShDetails(%22ctl00_ctl00_FindField_FindField_historyControl_ctrlPopup%22,%20%22S3%22,%20true);)  [Edit](https://web-p-ebscohost-com.ezp-prod1.hul.harvard.edu/Legacy/Views/UserControls/EHOST/) |
|  | S2 | (MH "Cannabis") OR (MH "Inhalant Abuse") | Expanders - Apply equivalent subjects  Search modes - Proximity | [View Results](javascript:__doPostBack('ctl00$ctl00$FindField$FindField$historyControl$HistoryRepeater$ctl12$linkResults','')) (12,471)  [View Details](javascript:showShDetails(%22ctl00_ctl00_FindField_FindField_historyControl_ctrlPopup%22,%20%22S2%22,%20true);)  [Edit](https://web-p-ebscohost-com.ezp-prod1.hul.harvard.edu/Legacy/Views/UserControls/EHOST/) |
|  | S1 | (MH "Substance Use Disorders") OR (MH "Persons with Substance Use Disorders") OR (MH "Substance Dependence") OR (MH "Substance Abuse") | Expanders - Apply equivalent subjects  Search modes - Proximity | [View Results](javascript:__doPostBack('ctl00$ctl00$FindField$FindField$historyControl$HistoryRepeater$ctl13$linkResults','')) (93,921)  [View Details](javascript:showShDetails(%22ctl00_ctl00_FindField_FindField_historyControl_ctrlPopup%22,%20%22S1%22,%20true);)  [Edit](https://web-p-ebscohost-com.ezp-prod1.hul.harvard.edu/Legacy/Views/UserControls/EHOST/) |

**(5) Web of Science, core collection (Clarivate)**

(“Drug Dependence” NOT “alcohol*”) OR ((cannabis OR cocaine OR crack OR hallucinogen OR hash* OR heroin OR inhalant? OR methamphetamine* OR psilocybin OR LSD OR meth OR marijuana OR multidrug OR phencyclidine OR polydrug OR polysubstance) “NEAR” (“use” OR “user?” OR using OR used))

OR

(“Drug Dependence” NOT “alcohol*”) OR ((cannabis OR cocaine OR crack OR hallucinogen OR hash* OR heroin OR inhalant? OR methamphetamine* OR psilocybin OR LSD OR meth OR marijuana OR multidrug OR phencyclidine OR polydrug OR polysubstance) “NEAR” (“abuse* OR abusing OR addict* OR depend* OR misuse? OR disorder? OR overuse? OR habit* OR rehab* OR recovery OR detox* OR “chemical’ depend*))

AND

((“self help” OR “twelve step”) OR (narcotics OR meth OR methamphetamine* OR cannabis OR marijuana OR cocaine OR methadone OR suboxone) “NEAR” (“anonymous” OR “support group” OR “peer support” OR “group therapy” OR “therap* group?” OR “recovery communit*” OR “community support” OR “support* community” OR “peer support”) “NEAR” (“recovery” OR “clean living” OR sober OR sobriety OR “drug free” OR “substance free” OR “detox”))

OR

(“virtual na” OR “na meeting?” OR “12 step” OR “twelve step?” OR “celebrate recovery” OR “lifering” OR “refugee recovery” OR “recovery dharma” OR “secular recovery” OR “secular sobriety” OR “secular organization*” OR “smart recovery” OR “community recovery” OR ”mutual aid” OR “mutual help” OR “self-management in recovery” OR “mutual support” OR (“web” OR “online” “NEAR” “forum”))
